# Divergent Picornavirus from a Turkey with Gastrointestinal Disease

**DOI:** 10.1128/genomeA.00134-13

**Published:** 2013-05-02

**Authors:** Terry Fei Fan Ng, Andrew K. Cheung, Walt Wong, Kelly M. Lager, Nikola O. Kondov, Yunhee Cha, Duane A. Murphy, Roman M. Pogranichniy, Eric Delwart

**Affiliations:** Blood Systems Research Institute, San Francisco, California, USAa;; Department of Laboratory Medicine, University of California—San Francisco, San Francisco, California, USAb;; Virus and Prion Diseases Research Unit, USDA, Agricultural Research Service, Ames, Iowa, USAc;; Indiana Animal Disease Diagnostic Laboratory and Department of Comparative Pathobiology, Purdue University, West Lafayette, Indiana, USAd

## Abstract

A novel picornavirus, turkey avisivirus (TuASV), was identified from the feces of turkeys (*Meleagris gallopavo*) with gastrointestinal disease from a farm in Indiana. Its genome organization is as follows: 5′ untranslated region (UTR)^IRES-II^ [VP0, VP3, VP1, 2A, 2B, 2C, 3A, 3B, 3C^pro^, 3D^pol^] 3′ UTR-poly(A). TuASV shares only 34% (P1), 36% (P2), and 35% (P3) amino acid identities with avihepatoviruses, indicating that it potentially represents a novel picornavirus genus.

## GENOME ANNOUNCEMENT

Picornaviruses are nonenveloped vertebrate-infecting viruses with a single linear positive-sense RNA genome (1). The family *Picornaviridae* consists of 12 formal genera, but potential new genera infecting humans and domestic and wild animals have been recently described (1; *Picornaviridae* website [http://www.picornaviridae.com]). Turkey production is an important food industry worldwide, valued at $5 billion in the United States in 2011 (2), but is susceptible to losses caused by infectious diseases. The recent discovery of two distinct picornavirus species in turkeys, turkey megrivirus from livers of turkeys with viral hepatitis in the United States (3) and turkey gallivirus from both healthy and sick turkeys from the United States and Hungary (4), has renewed the interest in studying turkey diseases.

In 2010, clinical disease presenting as fatigue and increased mortality was observed in 3- to 5-week-old domestic turkey poults from a breeding farm in Indiana. Diagnostic investigation revealed enteritis, and electron microscopy performed on gut contents detected picornavirus-like particles. In order to investigate the viral pathogens in these turkeys, viral metagenomics were performed on a pooled fecal sample from the sick birds using previously described protocols (5, 6). Sequences belonging to turkey gallivirus were identified, as well as novel viral sequences related (<50% amino acid identities) to the polyprotein from duck hepatitis A virus. The full genome of this new virus was acquired using reverse transcription-PCR (RT-PCR) and rapid amplification of cDNA ends (RACE).

The virus was provisionally named turkey avisivirus (turkey avihepatovirus sister-clade virus [TuASV-USA-IN1]) based on its relatedness to the duck hepatitis A virus of the genus *Avihepatovirus*, and for consistency with a publication in press describing a closely related virus named TuASV-1 (strain turkey/M176-TuASV/2011/HUN); TuASV-1 was found in Hungarian turkeys with stunting syndrome and/or poultry enteritis (7). TuASV-USA-IN1 and TuASV-1 share 83.5% nucleotide identities in the polyprotein open reading frame (ORF). The genome of strain TuASV-USA-IN1 was 7,373 bases long, with an overall base composition of 28.7% (A), 21.7% (C), 23.5% (G), and 26.1% (T). The genome organization was as follows: 5′ untranslated region (UTR)^IRES-II^[VP0, VP3, VP1, 2A, 2B, 2C, 3A, 3B, 3C^pro^, 3D^pol^] 3′ UTR-poly(A). The 5′ UTR was 609 bases long, containing a type II internal ribosome entry site (IRES-II), followed by a 6,495-base ORF (corresponding to 2,166 amino acids) encoding the polyprotein P1, P2, and P3 regions. The 3′ UTR was 266 bases long, followed by poly(A). Both 2A proteins (2A2 and 2A3) and the 2C protein contained the helicase nucleotide-binding motif GXXGXGK(T/S). Surprisingly, the closely related Hungarian viruses contained a third 2A protein (2A1) that is missing in the U.S. strain.

Strain TuASV-USA-IN1 shared 34% (P1), 36% (P2), and 35% (P3) amino acid identities with duck hepatitis virus (DHV) and lower identities with other picornaviruses. Based on the International Committee on Taxonomy of Viruses (ICTV) criteria, members of the same picornavirus genus share, for P1, P2, and P3 regions, >40%, 40%, and 50% amino acid identities, respectively. Therefore, TuASV-USA-IN1 likely reflects the presence of a new genus in the family *Picornaviridae*. As this is the first report of this virus in the United States, future studies are required to investigate its prevalence and pathogenicity in turkeys.

### Nucleotide sequence accession number.

The complete genome sequence of TuASV-USA-IN1 has been deposited in GenBank under accession no. KC614703.
